# Beyond risk management: reconsidering P-OCD in inpatient mental health care

**DOI:** 10.3389/fpsyt.2026.1937341

**Published:** 2026-09-15

**Authors:** Inga Piotrowska-Chen

**Affiliations:** The University of Manchester, Manchester, United Kingdom

**Keywords:** inpatient care, OCD, P-OCD, risk culture, risk management, safeguarding, stigma

## 1 Introduction

Obsessive–compulsive disorder (OCD) is characterised by recurrent intrusive thoughts, images or impulses (obsessions) and repetitive behaviours or mental acts (compulsions) (1). One of the key features distinguishing obsessions from non‑OCD worries is their ego‑dystonic nature: they feel contrary to the individual’s values and self‑concept, often generating intense anxiety and shame (2). In its most severe form, OCD can lead to significant self‑neglect, self‑harm, and even suicide attempts (3). OCD may also present as a co‑morbid condition in individuals with psychotic disorders, which can further complicate assessment and treatment (4). Despite this, OCD symptoms are not typically expected or routinely recognised on inpatient psychiatric units, meaning presentations may go unnoticed or misunderstood, with negative consequences for patient care (3). This paper focuses specifically on Pedophilia‑Themed OCD (P‑OCD) as an example of how taboo, ego‑dystonic intrusions may be misinterpreted within risk‑averse inpatient systems. Individuals with P-OCD present with intrusive, ego‑dystonic thoughts about harming children. These thoughts are profoundly distressing, and individuals often respond with compulsions such as mental reviewing, checking for signs of arousal, reassurance seeking, or avoiding children altogether (5). When these presentations are overlooked or misunderstood, clinicians are left managing distress and risk without a clear formulation, which intensifies a broader tension within inpatient settings: balancing patient‑centred care with the demands of a highly risk‑averse system (6).

## 2 Risk‑averse systems and their impact on OCD assessment

Behaviours and symptoms of P-OCD can superficially resemble those seen in Paedophilic Disorder, which involves ego-syntonic sexual interest in prepubescent children (7). The crucial distinction is function: in P-OCD, the person is trying to reduce anxiety and disconfirm feared harm; in paedophilic Disorder, avoidance may occur to prevent acting on sexual urges. The outward behaviours can look similar, but the internal drivers are fundamentally different. This nuance is easy to miss in busy inpatient environments, where staff must make rapid safeguarding decisions with limited information. Direct estimates of P-OCD misdiagnosis on general psychiatric inpatient wards are not currently available. However, evidence from other clinical settings indicates that atypical and taboo-themed presentations are frequently misidentified. In a vignette study of mental health professionals, 38.9% of OCD presentations were misidentified overall, including 42.9% of sexual obsessions involving children and 31.5% of aggressive obsessions (8). A study of primary-care physicians reported an overall misidentification rate of 50.5%, rising to 70.8% for paedophilia-themed obsessions (9). Stahnke’s (2021) systematic review similarly concluded that misdiagnoses and missed diagnoses of OCD were common across studies involving the general population, physicians and mental health practitioners, and noted that people with OCD may remain undiagnosed for ten years or longer. The review identified improved professional knowledge as an important priority. Although this evidence is not specific to inpatient settings, it demonstrates that symptom content can influence diagnostic recognition and strengthens the case for more reliable function-led differential assessment.

Inpatient care is organised around identifying and managing immediate, externally observable risks such as self‑harm, violence, or safeguarding concerns. Because of this focus, P-OCD presentations—especially those involving intrusive thoughts about harm—can be easily misinterpreted as indicators of actual risk by staff who are trained to detect genuine safeguarding issues. Given their safeguarding responsibilities, staff may be concerned about overlooking genuine risk and the professional consequences of doing so and therefore misinterpret the OCD symptoms as ego-syntonic. For example, if a patient with P-OCD starts compulsively confessing his intrusive thoughts to staff members, they might feel like they have to alert the safeguarding team and introduce special measures on the ward to decrease the risk of harm. That in turn can increase the patient’s guilt and embarrassment about their symptoms which are one of main drivers of OCD (10). In extreme scenarios, these risk-averse practices might, in rare cases, lead to social services involvement and children being taken away from their parents (11). These inpatient practices as well as lack of knowledge of OCD means that the more honest and help‑seeking the patient is, the more likely they are to be misunderstood, restricted, or even escalated within safeguarding pathways. In this sense, inpatient environments can inadvertently punish the very openness that effective OCD treatment requires. Wheaton et al. (12) found that self-concealment correlates with more severe OCD symptoms which might be because those individuals are less likely to seek support and therefore do not receive appropriate help.

These competing risks create a significant challenge for inpatient services: staff must respond cautiously to potentially serious disclosures, yet an exclusively content-led response can reinforce OCD, increase shame and concealment, and impede accurate assessment. The aim is therefore not to replace safeguarding with psychological formulation, but to integrate both so that immediate precautions remain proportionate, reviewable, and responsive to evidence about the function, context, and persistence of the thoughts.

## 3 Discussion

Risk-averse responses cannot be understood solely as a lack of psychological awareness. Inpatient staff often make rapid decisions with limited information, high-acuity caseloads, substantial safeguarding responsibilities and concern about the professional consequences of overlooking genuine risk. A content-led response may therefore appear safer, more defensible and easier to standardise than a nuanced functional assessment. Conversely, an overly confident assumption that distressing thoughts are ‘only OCD’ could delay appropriate safeguarding where intent, planning, access, behaviour or poor insight indicates genuine risk. Any proposed change must therefore improve assessment and review rather than lower the threshold for concern.

If inpatient teams are to distinguish genuine risk from ego‑dystonic distress, the system must equip them with clearer guidance and more psychologically informed frameworks. The existing differential-assessment approaches already emphasise evaluating ego-dystonicity, intent, behavioural evidence and the function of thoughts rather than their content alone (5, 7). National Institute for Health and Care Excellence guideline CG31 (13) addresses the recognition, assessment and treatment of OCD and recommends access to inpatient services with specific expertise in OCD. In practice, however, specialist provision remains limited, and many people with OCD are admitted to general psychiatric wards because of severe risk, difficulty engaging with outpatient treatment, or co-occurring mental health and psychosocial problems. Brakoulias et al. (3) emphasise that inpatient care frequently requires staff support, comprehensive risk assessment, treatment of comorbidities and attention to psychosocial difficulties alongside OCD-specific interventions. Yet current guidance provides limited operational detail for general ward teams responding when taboo-themed, ego-dystonic intrusions resemble safeguarding or violence-related risk.

Regular, multidisciplinary staff training is needed to encourage function rather than content-led assessments. The evidence base remains limited. However, Glazier & McGinn (14) asked psychology doctoral students to diagnose vignettes, and then presented them with a video intervention, increasing awareness of OCD symptoms. After exposure to the video, misdiagnosis of OCD rate dropped from 18.5% to 5.4% which suggests that targeted training could help develop a more accurate assessment process. The training should focus on distinguishing between the content and function of an individual’s thoughts. When clinicians focus primarily on what a person is thinking rather than why they are disclosing it, intrusive thoughts may be mistaken for ego-syntonic beliefs or intentions. Furthermore, the difference between ego-dystonic and ego-syntonic thoughts and recognising the role of inflated sense of responsibility and shame in OCD should be emphasised (15). However, providing and attending such trainings on busy, often-understaffed wards can be difficult to accomplish. Brief case-based sessions embedded within existing ward teaching may be more feasible than stand-alone programmes.

Increased involvement of psychological professionals may further support staff in responding to complex OCD presentations. Psychologists would be able to join multidisciplinary team meetings, presenting psychological formulations and encouraging staff to reflect on different presentations. Well-established psychology teams can offer rapid formulation support, modelling curiosity, and validating staff perspectives while also advocating for patients. Even so, it is important to remember that many wards lack psychological professionals due to funding restrictions. This presents yet another hurdle to overcome while implementing changes, with services potentially needing to consider alternative consultation arrangements. In settings with limited psychology provision, a designated staff member could receive additional training in OCD assessment and act as a liaison between the ward team and psychology services, facilitating access to specialist consultation when required.

Staff may also benefit from concise written guidance embedded within existing risk-assessment procedures. Such guidance could prompt consideration of ego-dystonicity, intent, planning, behavioural evidence and review of restrictions. Rather than introducing a new assessment framework, it would operationalise existing risk assessment principles within inpatient safeguarding processes to consider OCD-related presentations.

P-OCD provides a useful illustration of how taboo-themed obsessions can lead to tensions between risk management and formulation-led care in inpatient settings. The challenge discussed throughout this paper is not the absence of assessment principles, but the difficulty of translating them into day-to-day inpatient practice. Despite the existing guidance and various assessment approaches, OCD remains one of the most misdiagnosed mental health disorders generally, let alone in in-patient settings (16). This paper presents several practical suggestions on how the existing guidance could be implemented on psychiatric wards, while also acknowledging systemic barriers, such as understaffing, limited to access to specialist expertise and workforce pressures. These recommendations do not seek to reduce safeguarding vigilance. Rather, they aim to support decisions that are transparent, reviewable and informed by both risk assessment and psychological formulation, so that genuine risk is not overlooked while ego-dystonic distress is not inadvertently treated as evidence of intent.

Future research should investigate the prevalence of OCD misdiagnosis within general psychiatric inpatient settings. The impact of brief staff-training interventions, structured assessment guidance, and access to psychological consultation on diagnostic accuracy should also be evaluated. Patient outcomes would help determine which approaches are most effective in supporting formulation-led care without compromising safety.
